# Predictive value of the stress hyperglycemia ratio in dialysis patients with acute coronary syndrome: insights from a multi-center observational study

**DOI:** 10.1186/s12933-023-02036-7

**Published:** 2023-10-27

**Authors:** Enmin Xie, Zixiang Ye, Yaxin Wu, Xuecheng Zhao, Yike Li, Nan Shen, Yanxiang Gao, Jingang Zheng

**Affiliations:** 1 Department of Cardiology, China-Japan Friendship Hospital, 2 Yinghua Dongjie, Beijing, 100029 China; 2 China-Japan Friendship Hospital (Institute of Clinical Medical Sciences), Chinese Academy of Medical Sciences, Peking Union Medical College, Beijing, China; 3 Department of Cardiology, Peking University China-Japan Friendship School of Clinical Medicine, Beijing, China; 4 Department of Cardiology, Henan Provincial People’s Hospital, Fuwai Central China Cardiovascular Hospital, Zhengzhou, China

**Keywords:** Stress hyperglycemia, Acute coronary syndrome, Dialysis, Clinical outcomes

## Abstract

**Background:**

Various studies have indicated that stress hyperglycemia ratio (SHR) can reflect true acute hyperglycemic status and is associated with poor outcomes in patients with acute coronary syndrome (ACS). However, data on dialysis patients with ACS are limited. The Global Registry of Acute Coronary Events (GRACE) risk score is a well-validated risk prediction tool for ACS patients, yet it underestimates the risk of major events in patients receiving dialysis. This study aimed to evaluate the association between SHR and adverse cardiovascular events in dialysis patients with ACS and explore the potential incremental prognostic value of incorporating SHR into the GRACE risk score.

**Methods:**

This study enrolled 714 dialysis patients with ACS from January 2015 to June 2021 at 30 tertiary medical centers in China. Patients were stratified into three groups based on the tertiles of SHR. The primary outcome was major adverse cardiovascular events (MACE), and the secondary outcomes were all-cause mortality and cardiovascular mortality.

**Results:**

After a median follow-up of 20.9 months, 345 (48.3%) MACE and 280 (39.2%) all-cause mortality occurred, comprising 205 cases of cardiovascular death. When the highest SHR tertile was compared to the second SHR tertile, a significantly increased risk of MACE (adjusted hazard ratio, 1.92; 95% CI, 1.48–2.49), all-cause mortality (adjusted hazard ratio, 2.19; 95% CI, 1.64–2.93), and cardiovascular mortality (adjusted hazard ratio, 2.70; 95% CI, 1.90–3.83) was identified in the multivariable Cox regression model. A similar association was observed in both diabetic and nondiabetic patients. Further restricted cubic spline analysis identified a J-shaped association between the SHR and primary and secondary outcomes, with hazard ratios for MACE and mortality significantly increasing when SHR was > 1.08. Furthermore, adding SHR to the GRACE score led to a significant improvement in its predictive accuracy for MACE and mortality, as measured by the C-statistic, net reclassification improvement, and integrated discrimination improvement, especially for those with diabetes.

**Conclusions:**

In dialysis patients with ACS, SHR was independently associated with increased risks of MACE and mortality. Furthermore, SHR may aid in improving the predictive efficiency of the GRACE score, especially for those with diabetes. These results indicated that SHR might be a valuable tool for risk stratification and management of dialysis patients with ACS.

**Supplementary Information:**

The online version contains supplementary material available at 10.1186/s12933-023-02036-7.

## Background

Cardiovascular disease is the leading cause of death [1]. Patients with acute coronary syndrome (ACS) have a wide array of clinical manifestations and possess varying degrees of risk for experiencing adverse cardiovascular outcomes, making them a heterogeneous population [2, 3]. Current guidelines recommend utilizing risk stratification to identify high-risk patients for guidance in the triage and management of ACS patients [4, 5]. The Global Registry of Acute Coronary Events (GRACE) risk score is a well-validated risk prediction model in ACS patients, yet some validated predictors indicating poor prognosis have not been integrated into the tool [6]. Cardiovascular diseases affect over two-thirds of dialysis patients, and account for over half of all deaths in this population, with ACS playing a significant role in this disease spectrum [7, 8]. GRACE investigators have acknowledged the underestimation of risk by the GRACE score in dialysis patients with ACS, [9] necessitating further research combining prognostic variables with the GRACE score for accurate prognosis evaluation and refined risk stratification in dialysis patients with ACS.

Stress hyperglycemia is a condition characterized by the increase in glucose levels due to illness-related stress, [10, 11] which has been identified as a significant predictor for adverse events in ACS patients [12–14]. Adverse effects stemming from stress hyperglycemia can be attributed to a variety of mechanisms, such as inducing endothelial dysfunction and oxidative stress [11, 12]. Despite the significance of admission blood glucose (ABG) as a vital indicator of the acute hyperglycemic state, it cannot entirely mirror the state, since it may be affected by chronic glucose levels [10–12]. Therefore, Robert et al. proposed a new metric, the stress hyperglycemia ratio (SHR), that adjusts admission glucose concentration for background glycemia estimated from glycated hemoglobin (HbA1c) [15]. Several studies have demonstrated that SHR is a more reliable predictor of adverse outcomes in ACS patients than ABG [16–18]. However, dialysis patients have typically been excluded from these studies. Extremely high and low glucose levels have been associated with unfavorable outcomes in dialysis patients, including increased mortality risk [19, 20]. The utilization of SHR, following the calibration of admission glucose concentration with background glycemia, may provide a precise indicator of the acute glycemic surge [11, 15–18]. Nevertheless, the association between SHR and adverse outcomes in dialysis patients with ACS remains unexplored. Additionally, it is unknown whether adding the SHR to the GRACE score improves the risk stratification of dialysis patients with ACS.

Therefore, this study aimed to evaluate the association of SHR with adverse cardiovascular events in dialysis patients with ACS and explore the potential incremental prognostic value of incorporating SHR into the GRACE risk score.

## Methods

### Study design and population

**Fig. 1:**
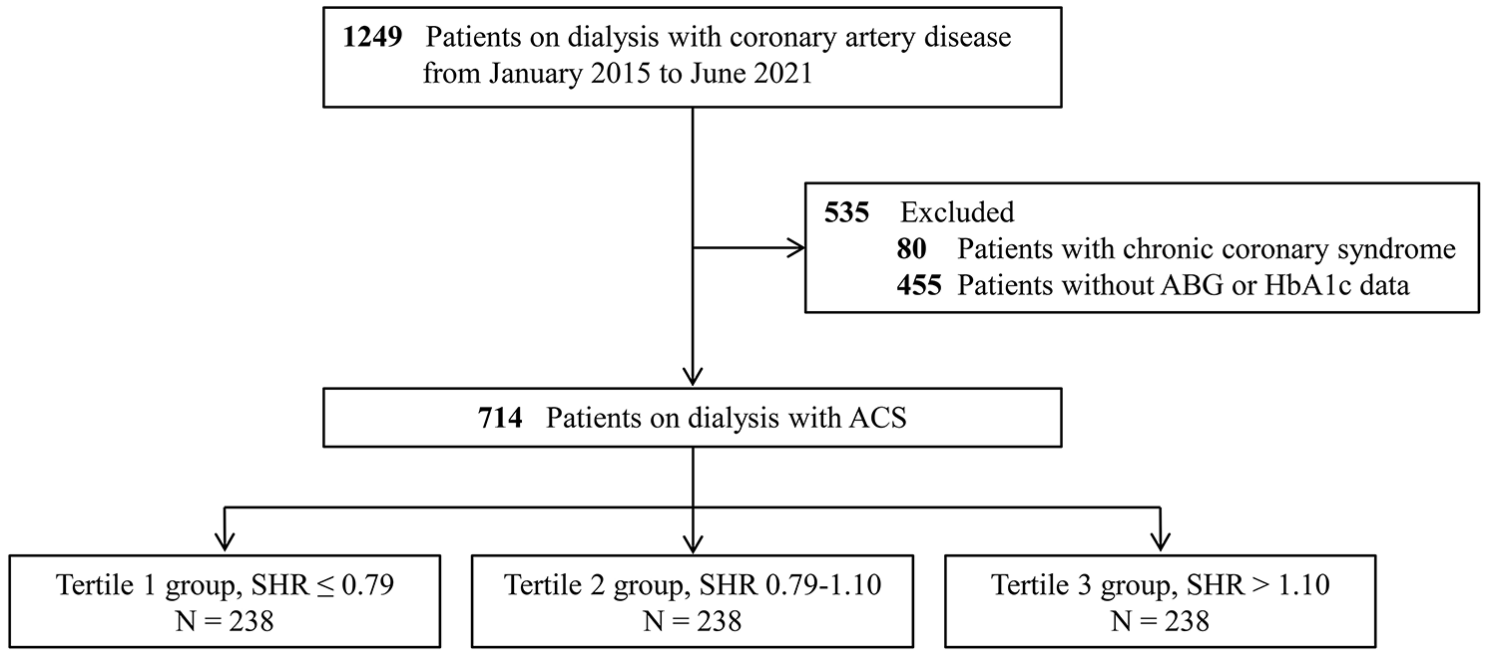
Study Flowchart. ABG = admission blood glucose; ACS = acute coronary syndrome; SHR = stress hyperglycemia ratio

The present study employed data obtained from the CRUISE-R (Coronary Revascularization in Patients On Dialysis in China-Retrospective) cohort study (ClinicalTrials.gov NCT05841082). The CRUISE-R study sought to undertake a comprehensive, observational, and multi-center registry investigation in China, targeting dialysis patients who were afflicted with coronary artery disease. The study’s principal objectives were to scrutinize the clinical features, therapeutic approaches, and prognosis of this particular patient population. A standardized protocol for patient selection was established with specific criteria and procedures, ensuring a uniform approach across all participating centers. The necessary information for screening criteria was easily available in electronic health records. All investigators involved in patient recruitment received unified training on the standardized screening criteria to reduce inter-center variability. Timely meetings and communication channels were established to address questions or concerns. Between January 2015 and June 2021, a total of 455,617 cardiac catheterizations were retrospectively evaluated in 30 tertiary medical centers, which were distributed across 12 provinces in China (Additional file 1: eAppendix 1). Exclusion criteria were rigorously applied, including patients who did not receive dialysis therapy or received dialysis therapy for less than 3 months (n = 453,421), those without any coronary stenosis exceeding 50% (n = 328), and individuals with other indications for coronary angiography (such as surgical interventions, valve diseases, or kidney transplants) (n = 87). For readmitted patients, only data from their initial admission were analyzed, with subsequent readmissions documented as “readmission” events (n = 532). Overall, the CRUISE-R study enlisted 1,249 dialysis patients suffering from obstructive coronary artery disease. The CRUISE-R registry study was carried out following the Declaration of Helsinki and was approved by local institutional review boards, with a waiver of informed consent. In the present analysis, we additionally eliminated 80 individuals with a diagnosis of stable angina and 455 patients with incomplete data on ABG or HbA1c. (Fig. 1) This study adhered to the Strengthening the Reporting of Observational Studies in Epidemiology (STROBE) statement.

### Data collection and definitions

The data were obtained from electronic medical records at each participating center by qualified study coordinators. The data set included patient demographics, comorbid conditions, cardiac history, cardiac function, location, and severity of coronary artery disease, procedure information, medical treatment, and clinical events. Key variables, including diagnosis, dialysis details, coronary severity, medical treatment, and clinical events, were inputted using double-entry by two trained researchers, with a third researcher verifying inconsistent data, while other variables underwent random audits. These validations ensured data accuracy and adherence to the screening criteria. A diagnosis of diabetes was deemed applicable when the individual had previously been diagnosed with the condition, was currently or previously using oral hypoglycemic drugs or insulin, or possessed HbA1c levels ≥ 6.5% at admission [21]. The definition of hypertension was based on the following criteria: systolic blood pressure ≥ 140 mmHg, diastolic blood pressure ≥ 90 mmHg, or the utilization of antihypertensive treatment [22]. The ABG was defined as the initial random serum glucose measurement taken within 24 h of hospital admission. Blood samples for HbA1c testing were also collected within 24 h of admission. The SHR was determined by dividing the glucose levels upon admission by the estimated average chronic glycemic value. The estimated average chronic glycemic level was obtained through the following equation: estimated average glucose (mmol/L) = 1.59 × HbA1c (%) − 2.59 [23]. In accordance with this, the formula for calculating the SHR was as follows: SHR = ABG/ [1.59 × HbA1c (%) − 2.59] [15]. The readily available hospital admission records furnished the necessary information to derive the GRACE risk scores, which run on a scale of 1 to 372 [6]. With higher GRACE scores indicating more precarious outcomes, the variables which were considered include age, heart rate, systolic blood pressure readings, creatinine levels, Killip classification, cardiac arrest during admission, the presence of ST-segment deviation, as well as cardiac biomarker levels.

### Outcomes

The primary outcome was major adverse cardiovascular events (MACE), as defined by a composite of all-cause death, non-fatal myocardial infarction, and non-fatal stroke. Myocardial infarction was confirmed through the presence of ischemic symptoms, elevated levels of serum cardiac biomarkers, and/or distinct ECG changes in patients. Stroke was confirmed as a new neurological deficit attributed to a vascular cause in the central nervous system, supported by imaging evidence from computed tomography or magnetic resonance imaging. Secondary outcomes were all-cause mortality and cardiovascular mortality. Cardiovascular mortality was defined as death due to acute myocardial infarction, heart failure, sudden cardiac death, stroke, cardiovascular procedure, or cardiovascular hemorrhage. Survival and clinical assessment data were gathered by trained nurses via outpatient clinic visits and telephone interviews. The follow-up period lasted until 30th June 2022 and was augmented by data extracted from the medical records of patients who were readmitted. Patients who were unavailable for telephone interviews were censored, and their survival status was fixed at the latest validated point in time, such as the date of the last outpatient clinic visit, or the final day of any hospital admission. Multiple occurrences of events were only evaluated once, with only the primary event included in the statistical analyses.

### Statistical analysis

Patients were stratified into three groups based on the SHR tertiles. Continuous variables are given as mean (standard deviation) or as the median and interquartile range (25th to 75th percentile) and analyzed with the ANOVA test or the Kruskal-Wallis H test as appropriate. Categorical variables are presented as frequency (percentages) and analyzed with the chi-square test or the Fisher exact test where appropriate.

The cumulative incidence of clinical outcomes was ascertained via the application of the Kaplan-Meier method and was compared with the log-rank test. The association between the SHR and primary and secondary outcomes was assessed with univariable and multivariable Cox proportional hazard models. For each outcome and exposure (SHR as either a continuous variable or tertiles), three models were implemented. Model 1 was an unadjusted analysis. Model 2 was adjusted for age and gender. Additionally, a completely adjusted Model 3 was analyzed, which was further adjusted for candidate variables listed in Table 1. Confounders that were significant in the univariate model, or of clinical importance, were included in Model 3. Results were reported as the hazard ratio (HR) and a 95% confidence interval (CI). The Schoenfeld residuals were used to ensure the proportional hazards assumption was met. Moreover, we conducted restricted cubic spline (RCS) analyses with five knots, placed at the 5th, 27.5th, 50th, 72.5th, and 95th centiles, to investigate potential nonlinear relationships between SHR and outcomes. Additionally, the RCS model was also adjusted for confounding factors included in Model 3. Incremental predictive performance after introducing ABG or SHR to the GRACE score was evaluated by calculating the C statistic, continuous net reclassification improvement (NRI), and integrated discrimination improvement (IDI) [24, 25]. Subgroup analyses were conducted based on diabetes status, with further analyses to assess the association between SHR and improvements in GRACE risk score predictive efficiency.

**Table 1:** Baseline demographic and clinical data of the study patients according to tertiles of stress hyperglycemia ratio

Characteristic	Overall *N* = 714	SHR1 (SHR ≤ 0.79) *N* = 238	SHR2 (0.79 < SHR ≤ 1.10) *N* = 238	SHR3 (SHR > 1.10) *N* = 238	*P* value
Age, mean (SD), yrs	62.0 (10.4)	61.4 (11.2)	61.4 (9.5)	63.1 (10.4)	0.109
Male, No. (%)	535 (74.9)	181 (76.1)	179 (75.2)	175 (73.5)	0.812
SBP, mean (SD), mmHg	141.6 (24.9)	141.7 (24.2)	144.7 (24.0)	138.3 (26.1)	0.020
DBP, mean (SD), mmHg	78.3 (13.4)	77.7 (12.7)	79.9 (13.9)	77.3 (13.4)	0.066
Heart rate, mean (SD), beats/min	80.4 (15.0)	77.6 (14.7)	79.8 (12.4)	83.6 (17.1)	< 0.001
Medical history and risk factors, No. (%)					
Hypertension	661 (92.6)	214 (89.9)	227 (95.4)	220 (92.4)	0.075
Diabetes mellitus	450 (63.0)	132 (55.5)	158 (66.4)	160 (67.2)	0.012
Current smoker	127 (17.8)	48 (20.2)	49 (20.6)	30 (12.6)	0.037
Atrial fibrillation	63 (8.8)	17 (7.1)	21 (8.8)	25 (10.5)	0.434
Cerebrovascular disease	139 (19.5)	39 (16.4)	45 (18.9)	55 (23.1)	0.174
Valvular disease	24 (3.4)	8 (3.4)	7 (2.9)	9 (3.8)	0.879
Peripheral arterial disease	74 (10.4)	22 (9.2)	25 (10.5)	27 (11.3)	0.751
Previous myocardial infarction	103 (14.4)	40 (16.8)	33 (13.9)	30 (12.6)	0.408
Previous intervention, No. (%)					
PCI	144 (20.2)	58 (24.4)	44 (18.5)	42 (17.6)	0.138
CABG	12 (1.7)	5 (2.1)	4 (1.7)	3 (1.3)	0.775
Dialysis modality, No. (%)					0.010
Hemodialysis	655 (91.7)	225 (94.5)	222 (93.3)	208 (87.4)	
Peritoneal dialysis	59 (8.3)	13 (5.5)	16 (6.7)	30 (12.6)	
Vintage, yrs					0.163
<1	151 (21.1)	39 (16.4)	62 (26.1)	50 (21.0)	
1–5	339 (47.5)	119 (50.0)	112 (47.1)	108 (45.4)	
5–10	183 (25.6)	68 (28.6)	50 (21.0)	65 (27.3)	
≥10	41 (5.7)	12 (5.0)	14 (5.9)	15 (6.3)	
Cause of dialysis, No. (%)					0.022
Diabetes mellitus	267 (37.4)	73 (30.7)	98 (41.2)	96 (40.3)	
Hypertension	87 (12.2)	39 (16.4)	25 (10.5)	23 (9.7)	
Glomerulonephritis	168 (23.5)	67 (28.2)	54 (22.7)	47 (19.7)	
Other/unknown	192 (26.9)	59 (24.8)	61 (25.6)	72 (30.3)	
Index presentation, No. (%)					0.037
AMI	450 (63.0)	137 (57.6)	149 (62.6)	164 (68.9)	
Unstable angina	264 (37.0)	101 (42.4)	89 (37.4)	74 (31.1)	
Hemoglobin, g/L	105.7 (19.4)	105.2 (20.1)	108.2 (19.3)	103.7 (18.5)	0.038
ABG,	8.0 (4.4)	5.1 (1.8)	7.5 (2.6)	11.5 (5.2)	< 0.001
HbA1c, %	6.5 (1.7)	6.7 (1.7)	6.7 (1.7)	6.3 (1.9)	0.011
Serum creatinine, mg/dl	8.9 (3.2)	9.5 (3.3)	8.8 (2.9)	8.6 (3.3)	0.007
GRACE score	157.0 [133.0, 182.0]	150.5 [130.0, 173.0]	155.5 [133.0, 181.0]	161.0 [139.2, 188.0]	0.001
Procedure characteristic, No. (%)					
Radial access	529 (74.1)	174 (73.1)	173 (72.7)	182 (76.5)	0.587
Extent of disease					
Any left main disease	91 (12.7)	32 (13.4)	30 (12.6)	29 (12.2)	0.916
2-vessel disease	187 (26.2)	55 (23.1)	57 (23.9)	75 (31.5)	0.072
≥3-vessel disease	426 (59.7)	150 (63.0)	144 (60.5)	132 (55.5)	0.231
Moderate or severe calcification	324 (45.4)	115 (48.3)	100 (42.0)	109 (45.8)	0.381
PCI treatment	512 (71.7)	174 (73.1)	173 (72.7)	165 (69.3)	0.604
Discharge medications, No. (%)					
Dual antiplatelet therapy	628 (88.0)	215 (90.3)	202 (84.9)	211 (88.7)	0.172
Aspirin	664 (93.0)	232 (97.5)	212 (89.1)	220 (92.4)	NA
Clopidogrel	596 (83.5)	200 (84.0)	195 (81.9)	201 (84.5)	NA
Ticagrelor	69 (9.7)	21 (8.8)	25 (10.5)	23 (9.7)	NA
ACE inhibitor or ARB	343 (48.0)	120 (50.4)	111 (46.6)	112 (47.1)	0.664
*β*-blocker	587 (82.2)	188 (79.0)	199 (83.6)	200 (84.0)	0.280
Calcium-channel blocker	467 (65.4)	161 (67.6)	163 (68.5)	143 (60.1)	0.105
Statin	678 (95.0)	228 (95.8)	223 (93.7)	227 (95.4)	0.541

Data are presented as mean (SD) or n (%)

ABG = admission blood glucose; ACE = angiotensin-converting enzyme; AMI = acute myocardial infarction; ARB = angiotensin receptor blocker; CABG = coronary artery bypass grafting; DBP = diastolic blood pressure; GRACE = Global Registry of Acute Coronary Events; PCI = percutaneous coronary intervention; SBP = systolic blood pressure; SHR = stress hyperglycemia ratio

Furthermore, in individuals with low admission hemoglobin values, there exists a potential for an underestimation of the average chronic glucose level calculated from HbA1c. Thus, a sensitivity analysis was conducted to assess the robustness of the main results by excluding patients with admission hemoglobin < 100 g/L. All *P* values were two-sided, and statistical significance was considered at a value of < 0.05. The data were analyzed using SPSS 23.0 (IBM SPSS 23 Inc) and R 3.6.1 (R Development Core Team, Vienna, Austria).

## Results

### Baseline characteristics

A total of 714 dialysis patients with ACS were recruited in the current study. The mean age was 62.0 (10.4) years. The majority of patients were male (74.9%), and a significant proportion had diabetes (63.0%). The median value of SHR for the entire cohort was 0.91 (0.74–1.23). Based on the tertiles of SHR, patients were divided into three groups: SHR1 (SHR ≤ 0.79), SHR2 (0.79 < SHR ≤ 1.10), and SHR3 (1.10 < SHR). The highest tertile (SHR3 group) comprised a greater proportion of patients who suffered from diabetes mellitus, received peritoneal dialysis, and had presented with acute myocardial infarction. Additionally, lower systolic blood pressure, elevated heart rate, reduced hemoglobin and serum creatinine levels, and higher GRACE scores were observed more frequently in the SHR3 group. A detailed summary of baseline and clinical characteristics is presented in Table 1. Furthermore, Table S1 (Additional file 1) presents a comparison between the final population and the population that was excluded due to missing ABG or HbA1c measurements.

### Outcomes

**Fig. 2:**
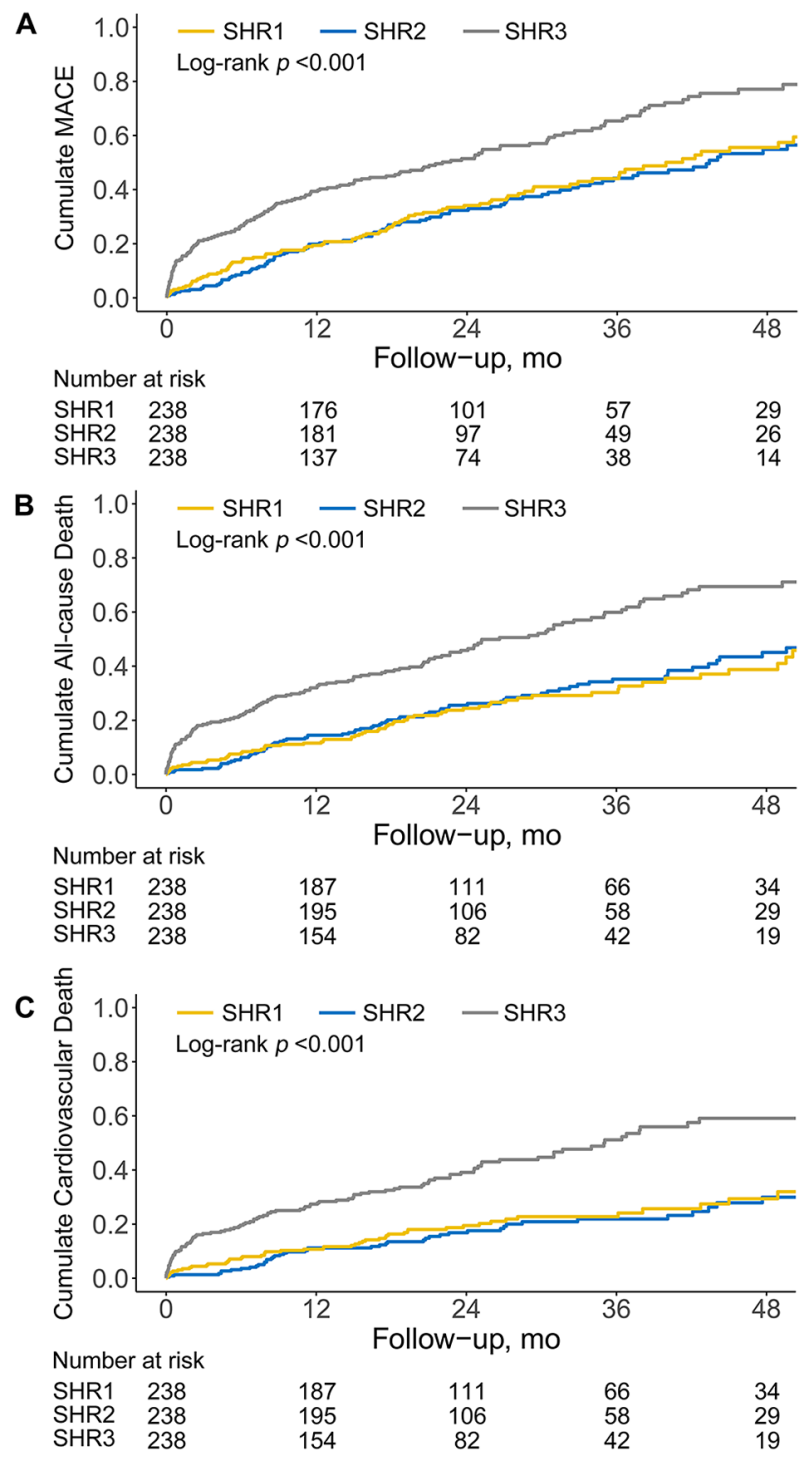
Kaplan–Meier curves for major adverse cardiovascular events **(A)**, all-cause mortality **(B)**, and cardiovascular mortality **(C)** according to tertiles of stress hyperglycemia ratio. MACE = Major Adverse Cardiovascular Events, SHR = stress hyperglycemia ratio

**Fig. 3:**
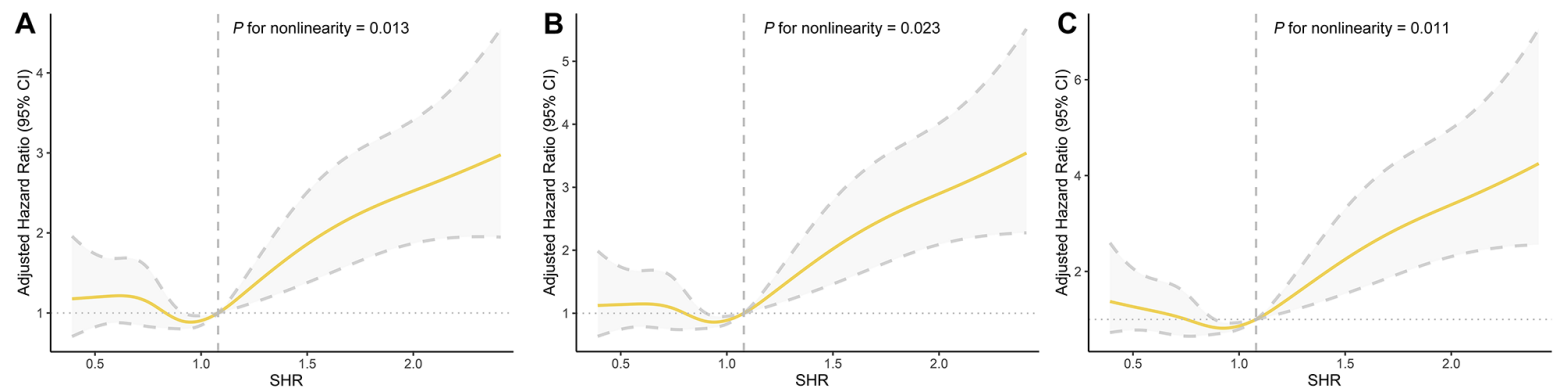
Multivariable restricted cubic spline regression showed the nonlinear association of stress hyperglycemia ratio with clinical outcomes after full adjustment (Model 3). A: SHR and MACE. B: SHR and all-cause mortality. C: SHR and cardiovascular mortality. Hazard ratios were indicated by yellow solid lines and 95% CIs by grey dotted lines. SHR = stress hyperglycemia ratio

The median duration of follow-up was 20.9 (12.0-34.5) months. A total of 345 MACE were observed, including 280 cases of all-cause mortality (comprising 205 cases of cardiovascular death), 69 cases of stroke, and 24 cases of nonfatal myocardial infarction (Additional file 1: Table S2). Figure 2 presents the Kaplan-Meier curves demonstrating the incidence of primary and secondary outcomes for the SHR tertiles. A statistically significant difference in the incidence of MACE, all-cause mortality, and cardiovascular mortality was identified among the three groups, with the highest incidence recorded in the SHR3 group. Cox regression analyses were conducted to identify independent risk factors and calculate the HRs of the SHR for clinical outcomes (Table 2). When the highest SHR tertile was compared to the second SHR tertile, a significantly increased risk of MACE (adjusted HR, 1.92; 95% CI, 1.48–2.49), all-cause mortality (adjusted HR, 2.19; 95% CI, 1.64–2.93), and cardiovascular mortality (adjusted HR, 2.70; 95% CI, 1.90–3.83) was identified in the fully adjusted model (Model 3; all *P* values < 0.001). Similarly, modeling SHR as a continuous variable revealed a significantly increased risk of MACE, all-cause mortality, and cardiovascular mortality with higher SHR levels among the patients (Table 2). Moreover, the RCS analyses revealed non-linear and J-shaped associations between the SHR and MACE, all-cause mortality, and cardiovascular mortality, after full adjustment (all *P* values for nonlinearity < 0.05) (Fig. 3). An inflection point of 1.08 was observed for each endpoint, indicating that when the SHR remained below 1.08, the HR experienced a modest change, and the 95% CI of the HR encompassed 1. Conversely, a significant and sudden escalation in HR was recorded when the SHR exceeded 1.08.

**Table 2:** Associations between stress hyperglycemia ratio and clinical outcomes

	Model 1				Model 2				Model 3		
	HR	95% CI	*P* value		HR	95% CI	*P* value		HR	95% CI	*P* value
MACE^a^											
SHR index	1.89	1.54–2.32	< 0.001		1.85	1.50–2.27	< 0.001		1.86	1.51–2.30	< 0.001
SHR1	1.09	0.82–1.44	0.562		1.09	0.83–1.45	0.531		1.07	0.81–1.42	0.634
SHR2	Reference				Reference				Reference		
SHR3	1.98	1.53–2.56	< 0.001		1.94	1.50–2.51	< 0.001		1.92	1.48–2.49	< 0.001
All-cause Mortality^b^											
SHR index	2.31	1.87–2.87	< 0.001		2.25	1.81–2.79	< 0.001		2.20	1.76–2.77	< 0.001
SHR1	0.93	0.68–1.29	0.679		0.94	0.68–1.29	0.686		1.05	0.75–1.46	0.787
SHR2	Reference				Reference				Reference		
SHR3	2.17	1.64–2.86	< 0.001		2.11	1.59–2.79	< 0.001		2.19	1.64–2.93	< 0.001
Cardiovascular Mortality^c^											
SHR index	2.55	2.00-3.25	< 0.001		2.49	1.95–3.18	< 0.001		2.48	1.92–3.20	< 0.001
SHR1	1.13	0.77–1.68	0.529		1.14	0.77–1.68	0.517		1.18	0.79–1.77	0.409
SHR2	Reference				Reference				Reference		
SHR3	2.71	1.93–3.81	< 0.001		2.65	1.88–3.73	< 0.001		2.70	1.90–3.83	< 0.001

Model 1: Unadjusted. Model 2: Adjusted for age and sex

^a^Model 3 for MACE: Adjusted for age, sex, SBP, DBP, AMI as index presentation, GRACE score, left main disease, 3-vessel disease, moderate or severe calcification, dual antiplatelet therapy, ACE inhibitor or ARB, and calcium-channel blocker

^b^Model 3 for all-cause mortality: Adjusted for age, sex, heart rate, SBP, DBP, diabetes mellitus, current smoker, AMI as index presentation, serum creatinine, GRACE score, left main disease, 3-vessel disease, moderate or severe calcification, PCI treatment, ACE inhibitor or ARB, and calcium-channel blocker

^c^Model 3 for cardiovascular mortality: Adjusted for age, sex, SBP, DBP, dialysis vintage, AMI as index presentation, GRACE score, left main disease, 3-vessel disease, moderate or severe calcification, PCI treatment, ACE inhibitor or ARB, and calcium-channel blocker

ACE = angiotensin-converting enzyme; AMI = acute myocardial infarction; ARB = angiotensin receptor blocker; CI = confidence interval; DBP = diastolic blood pressure; GRACE = Global Registry of Acute Coronary Events; HR = hazard ratio; MACE = Major Adverse Cardiovascular Events; PCI = percutaneous coronary intervention; SBP = systolic blood pressure; SHR = stress hyperglycemia ratio

### Incremental predictive value of adding SHR to the GRACE score

Overall, the inclusion of SHR significantly enhanced risk predictions for primary and secondary outcomes when compared to the use of the GRACE risk score in the entire cohort (Table 3). The addition of SHR to the GRACE risk score resulted in significant improvements in C-statistics for the prediction of MACE, all-cause mortality, and cardiovascular mortality, with changes of 0.028 (95% CI, 0.007–0.050), 0.041 (95% CI, 0.015–0.066) and 0.046 (95% CI, 0.015–0.078), respectively. Modeling as a categorical variable (> 1.08) demonstrated that SHR still improved the predictive capacity of the GRACE risk score. Additionally, significant enhancements in reclassification were noted as evaluated by the continuous NRI with 0.212 (95% CI, 0.116–0.303) for MACE, 0.216 (95% CI, 0.104–0.307) for all-cause mortality, and 0.256 (95% CI, 0.159–0.371) for cardiovascular mortality (all *P* values < 0.001). Similar results were obtained for IDI with a score of 0.028 (95% CI, 0.006–0.055) for MACE, 0.031 (95% CI, 0.006–0.065) for all-cause mortality, and 0.042 (95% CI, 0.012–0.081). Notably, the addition of ABG to the GRACE risk score did not result in any significant improvement in the C-statistic, NRI, or IDI for predicting primary and secondary outcomes.

**Table 3:** Added predictive ability and reclassification statistics of stress hyperglycemia ratio (SHR) as a continuous or categorical variable

	C-statistic (95% CI)	ΔC-statistic (95% CI)	*P* Value	Continuous NRI (95% CI)	*P* Value	IDI (95% CI)	*P* Value
**MACE**							
GRACE score	0.606 (0.573, 0.639)	Reference		Reference		Reference	
GRACE score + ABG	0.613 (0.580, 0.646)	0.006 (-0.003, 0.016)	0.174	0.092 (-0.067, 0.222)	0.259	0.005 (-0.002, 0.021)	0.289
GRACE score + SHR (as continuous variable)	0.635 (0.604, 0.666)	0.028 (0.007, 0.050)	0.010	0.128 (0.008, 0.249)	0.040	0.024 (0.001, 0.053)	0.040
GRACE score + SHR (> 1.08)	0.633 (0.602, 0.664)	0.027 (0.006, 0.048)	0.012	0.212 (0.116, 0.303)	< 0.001	0.028 (0.006, 0.055)	< 0.001
**All-cause mortality**							
GRACE score	0.629 (0.594, 0.664)	Reference		Reference		Reference	
GRACE score + ABG	0.643 (0.608, 0.678)	0.014 (0, 0.028)	0.052	0.135 (0.011, 0.255)	0.030	0.013 (0.000, 0.033)	0.070
GRACE score + SHR (as continuous variable)	0.670 (0.635, 0.705)	0.041 (0.015, 0.066)	0.002	0.154 (0.036, 0.277)	0.020	0.038 (0.005, 0.074)	< 0.001
GRACE score + SHR (> 1.08)	0.662 (0.627, 0.697)	0.033 (0.010, 0.057)	0.006	0.216 (0.104, 0.307)	< 0.001	0.031 (0.006, 0.065)	0.010
**Cardiovascular mortality**							
GRACE score	0.628 (0.587, 0.669)	Reference		Reference		Reference	
GRACE score + ABG	0.641 (0.600, 0.682)	0.013 (-0.003, 0.028)	0.104	0.146 (0.017, 0.299)	0.020	0.013 (0.000, 0.037)	0.040
GRACE score + SHR (as continuous variable)	0.674 (0.633, 0.715)	0.046 (0.015, 0.078)	0.004	0.239 (0.097, 0.357)	< 0.001	0.049 (0.010, 0.094)	< 0.001
GRACE score + SHR (> 1.08)	0.674 (0.635, 0.713)	0.046 (0.017, 0.076)	0.002	0.256 (0.159, 0.371)	< 0.001	0.042 (0.012, 0.081)	< 0.001

ABG = admission blood glucose; CI = confidence interval; GRACE = Global Registry of Acute Coronary Events; IDI = integrated discrimination improvement; NRI = net reclassification improvement; SHR = stress hyperglycemia ratio

### Subgroup analyses and sensitivity analyses

**Fig. 4:**
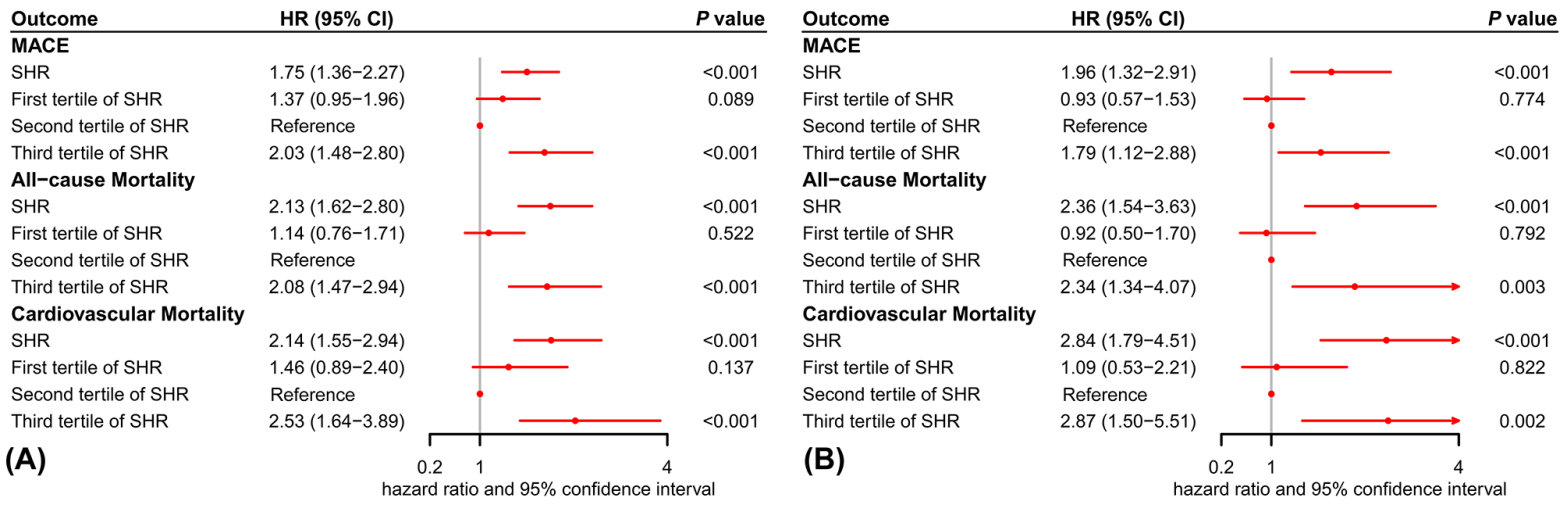
The adjusted hazards ratio of stress hyperglycemia ratio for clinical outcomes in the diabetic (A) and nondiabetic (B) patients. CI = confidence interval; HR = hazard ratio; SHR = stress hyperglycemia ratio

To evaluate the potential modified effect of diabetes status on the association between SHR and outcomes, as well as the incremental predictive value of adding SHR to the GRACE score, subgroup analyses were performed on individuals with and without diabetes. After adjusting for multiple potential confounding factors, the highest SHR tertile retained a significant association with an increased risk of MACE, all-cause mortality, and cardiovascular mortality in both diabetic and nondiabetic patients (Fig. 4). Furthermore, these results remained robust when SHR was modeled as a continuous variable (Fig. 4). RCS analyses conducted on diabetic patients also demonstrated a J-shaped relationship between SHR and the risk of MACE and mortality (all *P* values for nonlinearity < 0.05), while this relationship was not statistically significant for non-diabetic patients (Additional file 1: Figures S1-2). Additionally, verification of the incremental effect of adding SHR to the GRACE score for predicting primary and secondary outcomes was established in diabetic individuals as evidenced by an improvement in C-statistics, NRI, and IDI (Table 4). However, it should be noted that although there was an improvement in C-statistics, NRI and IDI analyses were insufficient in detecting a significant difference in the incremental effect of adding SHR to the GRACE score for predicting poor prognosis in nondiabetic individuals (Table 4).

**Table 4:** Discrimination and reclassification performance of stress hyperglycemia ratio (SHR) in predicting outcomes According to diabetes status

	C-statistic (95% CI)	ΔC-statistic (95% CI)	*P* Value	Continuous NRI (95% CI)	*P* Value	IDI (95% CI)	*P* Value
**Prediction for MACE in diabetic patients**							
GRACE score	0.597 (0.556, 0.638)	Reference		Reference		Reference	
GRACE score + ABG	0.601 (0.560, 0.642)	0.003 (-0.006, 0.012)	0.514	-0.019 (-0.185, 0.151)	0.891	0.003 (-0.002, 0.019)	0.396
GRACE score + SHR (as continuous variable)	0.622 (0.581, 0.663)	0.024 (-0.005, 0.054)	0.101	0.173 (0.011, 0.355)	0.030	0.030 (-0.002, 0.068)	0.090
GRACE score + SHR (> 1.08)	0.627 (0.588, 0.666)	0.030 (0.001, 0.059)	0.045	0.206 (0.071, 0.429)	< 0.001	0.061 (0.013, 0.129)	< 0.001
**Prediction for all-cause mortality in diabetic patients**							
GRACE score	0.612 (0.567, 0.657)	Reference		Reference		Reference	
GRACE score + ABG	0.625 (0.580, 0.670)	0.013 (-0.006, 0.032)	0.167	0.061 (-0.131, 0.215)	0.428	0.012 (-0.002, 0.039)	0.149
GRACE score + SHR (as continuous variable)	0.653 (0.608, 0.698)	0.042 (0.007, 0.076)	0.019	0.223 (0.047, 0.360)	< 0.001	0.043 (0.003, 0.099)	0.020
GRACE score + SHR (> 1.08)	0.651 (0.608, 0.694)	0.039 (0.007, 0.071)	0.017	0.218 (0.082, 0.342)	< 0.001	0.037 (0.004, 0.085)	< 0.001
**Prediction for cardiovascular mortality in diabetic patients**							
GRACE score	0.620 (0.567, 0.673)	Reference		Reference		Reference	
GRACE score + ABG	0.630 (0.577, 0.683)	0.01 (-0.010, 0.029)	0.325	0.076 (-0.130, 0.258)	0.388	0.013 (-0.002, 0.048)	0.169
GRACE score + SHR (as continuous variable)	0.664 (0.613, 0.715)	0.045 (0.004, 0.086)	0.033	0.267 (0.097, 0.430)	< 0.001	0.056 (0.002, 0.126)	0.040
GRACE score + SHR (> 1.08)	0.671 (0.622, 0.720)	0.052 (0.012, 0.091)	0.010	0.268 (0.129, 0.395)	< 0.001	0.052 (0.007, 0.116)	0.010
**Prediction for MACE in nondiabetic patients**							
GRACE score	0.620 (0.565, 0.675)	Reference		Reference		Reference	
GRACE score + ABG	0.635 (0.582, 0.688)	0.016 (-0.005, 0.036)	0.133	-0.064 (-0.213, 0.270)	0.871	0.002 (-0.009, 0.037)	0.634
GRACE score + SHR (as continuous variable)	0.649 (0.596, 0.702)	0.030 (-0.002, 0.061)	0.065	0.053 (-0.195, 0.266)	0.478	0.009 (-0.016, 0.052)	0.637
GRACE score + SHR (> 1.08)	0.640 (0.587, 0.693)	0.020 (-0.010, 0.049)	0.188	0.165 (-0.063, 0.333)	0.159	0.014 (-0.007, 0.075)	0.338
**Prediction for all-cause mortality in nondiabetic patients**							
GRACE score	0.659 (0.598, 0.720)	Reference		Reference		Reference	
GRACE score + ABG	0.678 (0.617, 0.739)	0.018 (-0.006, 0.042)	0.144	0.019 (-0.166, 0.140)	0.909	0.011 (-0.009, 0.022)	0.545
GRACE score + SHR (as continuous variable)	0.695 (0.636, 0.754)	0.035 (0, 0.071)	0.050	0.045 (-0.113, 0.331)	0.368	0.021 (-0.012, 0.091)	0.368
GRACE score + SHR (> 1.08)	0.682 (0.625, 0.739)	0.023 (-0.010, 0.057)	0.177	0.207 (-0.001, 0.407)	0.060	0.016 (-0.012, 0.086)	0.398
**Prediction for cardiovascular mortality in nondiabetic patients**							
GRACE score	0.642 (0.571, 0.713)	Reference		Reference		Reference	
GRACE score + ABG	0.670 (0.599, 0.741)	0.028 (-0.007, 0.063)	0.117	0.022 (-0.329, 0.277)	0.786	0.020 (-0.034, 0.111)	0.229
GRACE score + SHR (as continuous variable)	0.691 (0.622, 0.760)	0.049 (-0.001, 0.099)	0.054	0.111 (-0.135, 0.451)	0.289	0.030 (-0.022, 0.158)	0.358
GRACE score + SHR (> 1.08)	0.678 (0.611, 0.745)	0.036 (-0.008, 0.080)	0.107	0.231 (-0.134, 0.514)	0.129	0.021 (-0.054, 0.143)	0.418

ABG = admission blood glucose; CI = confidence interval; GRACE = Global Registry of Acute Coronary Events; IDI = integrated discrimination improvement; MACE = Major Adverse Cardiovascular Events; NRI = net reclassification improvement; SHR = stress hyperglycemia ratio

Considering the potential underestimation of average chronic glucose levels calculated from HbA1c in patients with low admission hemoglobin values, we excluded 263 patients with admission hemoglobin < 100 g/L. Multivariable Cox regression and RCS analyses also showed J-shaped associations between SHR and primary and secondary outcomes (Additional file 1: Figures S3-4). Moreover, incorporating SHR into the GRACE score continued to have an incremental effect on predicting MACE and mortality after excluding patients with admission hemoglobin < 100 g/L (Additional file 1: Table 3).

## Discussion

This multi-center study evaluated the prognostic potential of SHR in dialysis patients with ACS. Our results indicated that SHR was independently associated with an increased risk of MACE and mortality in dialysis patients with ACS, irrespective of diabetes status. These correlations manifest non-linearly, displaying a J-shaped pattern, with hazard ratios for MACE and mortality significantly increasing when SHR was > 1.08. Furthermore, our study provides evidence that adding SHR to the GRACE score could significantly improve the predictive value of MACE and mortality, particularly in those with diabetes.

Stress hyperglycemia is prevalent among ACS patients and is known to be a significant prognostic factor for short- and long-term outcomes [12–14]. Previous ABG-based studies have limitations in accurately assessing stress hyperglycemia due to the influence of both chronic glycemic state and acute stress conditions, particularly in patients with existing diabetes mellitus. SHR is a new metric that directly connects a patient’s present glucose level during the acute phase and their background glycemia, producing a personalized calculation of stress hyperglycemia [15]. Various studies have been conducted to investigate the prognostic value of SHR in patients with ACS. Yang et al. observed a significant association between SHR and early and late poor prognosis in 5562 patients with ACS who underwent the implantation of a drug-eluting stent [16]. Another study of 2089 patients with acute myocardial infarction (AMI) revealed that SHR was an independent predictor of long-term mortality, regardless of diabetes status [26]. Additionally, Xu et al. found that SHR was related to the risks of 30-day MACEs and mortality in patients with ST-segment–elevation myocardial infarction (STEMI) [27]. A further study discovered an elevated risk of long-term all-cause mortality in STEMI patients who underwent percutaneous coronary intervention (PCI) [28]. However, dialysis patients accounted for only a small percentage or were excluded from these studies, and there is little data to support an association between SHR and cardiovascular adverse events in dialysis patients with ACS.

The present study provided evidence of the independent association between SHR and adverse cardiovascular events, including MACE, all-cause mortality, and cardiovascular mortality in dialysis patients with ACS, regardless of their diabetes status. The correlation was found to be J-shaped by further RCS analysis, with HRs for MACE and mortality increasing significantly when SHR reached > 1.08. Notably, while diabetic subgroup analyses yielded results consistent with those of the overall population, the J-shaped association was not significant in nondiabetic patients. The findings of the current study are consistent with previous research in some regards. Yang et al. noted a J-shaped or U-shaped association between SHR and poor prognosis in ACS patients, with this association being non-significant in the subgroup of patients without diabetes, which corresponds with our findings [16]. Similarly, Wei et al. found a J-shaped relationship between SHR and in-hospital death and all-cause mortality in STEMI patients who received PCI treatment [28]. Additionally, a study involving 3750 AMI patients found that severe hyperglycemia and euglycemia were associated with higher mortality in patients with diabetes than moderate hyperglycemia levels. In contrast, this relationship was linear in non-diabetic patients, with lower glycemia levels being associated with the lowest mortality rates [29]. Furthermore, stress hyperglycemia, as measured by glucose/glycated albumin ratio, exhibited a significant U-shaped relationship only in ACS patients with diabetes mellitus, as opposed to those without diabetes, which partially aligns with our findings [17]. The mechanisms that connect SHR with adverse outcomes in ACS patients are not yet well understood, however, there may be several factors involved. One possible mechanism is a reduction in vasodilation that is dependent on endothelial function [30]. Additionally, SHR may be associated with impaired platelet anti-aggregatory effects [31]. Furthermore, hyperactivation of the sympathetic nervous system with a pro-inflammatory pathway may be involved in the negative health outcomes associated with SHR in ACS patients [32].

The GRACE risk score is a well-validated tool for risk stratification in ACS patients [4–6]. However, the GRACE registry investigators have acknowledged that the GRACE score could potentially underestimate the likelihood of adverse events in ACS patients undergoing dialysis [9]. In response, they have proposed conducting more research into the integration of biomarkers that would provide additional prognostic value, supplementing the existing GRACE score. Our study demonstrated that incorporating the SHR into the GRACE score improved its predictive accuracy for MACE, all-cause mortality, and cardiovascular mortality, as measured by an improvement in C-statistics, NRI, and IDI. Furthermore, the most significant advantages were noticed among patients with diabetes. In nondiabetic individuals, despite there being a significant improvement in C-statistics, NRI and IDI analyses were insufficient in detecting a significant difference in the incremental effect of adding SHR to the GRACE score for predicting poor prognosis. These results are consistent with the study of Luo et al., [26] in which adding SHR to the GRACE score resulted in better mortality prediction in AMI patients with diabetes compared to those without. Meanwhile, Xiong et al. demonstrated that adding SHR to the GRACE score could better identify ACS patients who are at a high risk of long-term adverse outcomes, regardless of diabetes status [33]. The discrepancy may be attributed to differences in study populations since the prior studies had underrepresented dialysis patients. Overall, our study supports the use of combining SHR with the GRACE score as an effective tool to stratify risk in dialysis patients with ACS, providing new insights for improving the clinical value of the GRACE risk score for this specific patient population.

### Strength and limitations

This study, to our knowledge, is the first to evaluate the prognostic potential of SHR in dialysis patients with ACS. Moreover, we propose a J-shaped association between SHR and a poor prognosis in dialysis patients with ACS and, for the first time, support the incremental prognostic value of incorporating SHR into the GRACE risk score in this particular patient population. Nonetheless, our results are subject to certain limitations. The retrospective nature of the study is noteworthy, as it raises concerns about the possible influence of confounding variables and selection bias on the outcomes. Furthermore, information pertaining to the duration of diabetes, treatment for glycemic control, and adherence to medication after discharge were not available in this study. Lastly, the relatively small size of the subgroup populations may have biased the findings. To overcome these limitations, more comprehensive data from broader studies with larger sample sizes and extended follow-up will be necessary to validate our findings and improve our understanding of the association between SHR and prognosis in dialysis patients with ACS.

## Conclusions

In summary, our study demonstrated that SHR was an independent predictor of MACE and mortality in dialysis patients with ACS, regardless of their diabetes status. Incorporating SHR might improve the predictive accuracy of the GRACE score in dialysis patients with ACS, especially those with diabetes. Therefore, SHR has the potential to be integrated into risk stratification strategies for dialysis patients with ACS, enabling more precise prognostic assessment and improved clinical management decisions. Further studies are needed to validate these findings and explore the underlying mechanisms.

### Electronic supplementary material

Below is the link to the electronic supplementary material.

Supplementary Material 1

## Data Availability

The dataset analyzed during the current study is available from the corresponding author on reasonable request.

## Abbreviations

ABG: Admission blood glucose
ACS: Acute coronary syndrome
AMI: Acute myocardial infarction
CI: Confidence interval
GRACE: Global Registry of Acute Coronary Events
HbA1c: Glycosylated hemoglobin
HR: Hazard ratio
IDI: Integrated discrimination improvement
MACE: Major adverse cardiovascular events
NRI: Net reclassification improvement
RCS: Restricted cubic spline
SHR: Stress hyperglycemia ratio
STEMI: ST-Segment–Elevation Myocardial Infarction
