# Supplementary material for: Predictive value of the stress hyperglycemia ratio in dialysis patients with acute coronary syndrome: insights from a multi-center observational study
Source: Cardiovasc Diabetol. 2023 Oct 27;22:288. doi: 10.1186/s12933-023-02036-7 (PMC10612265; doi:10.1186/s12933-023-02036-7)
Supplement: Supplementary file 1 — Supplementary Material 1 [file 12933_2023_2036_MOESM1_ESM.pdf]

SUPPLEMENTAL MATERIAL

eAppendix 1. List of Principal Investigators and Participating Centers

No.	Name	Affiliation	Region	Province
1	Jingang Zheng	China-Japan Friendship Hospital	North China	Beijing
2	Yong He	West China Hospital	Southwest China	Sichuan
3	Hesong Zeng	Tongji Hospital	Central China	Hubei
4	Jianfang Luo	Guangdong Provincial People’s Hospital	South China	Guangdong
5	Mulei Chen	Beijing Chaoyang Hospital	North China	Beijing
6	Wenyue Pang	Shengjing Hospital of China Medical University	Northeast China	Liaoning
7	Yanmin Xu	Second Hospital of Tianjin Medical University	North China	Tianjin
8	Chuanyu Gao	Fuwai Central China Cardiovascular Hospital	Central China	Henan
9	Xiaogang Guo	The First Affiliated Hospital, Zhejiang University School of Medicine	East China	Zhejiang
10	Lin Cai	The Third People's Hospital of Chengdu	Southwest China	Sichuan
11	Qingwei Ji	The People’s Hospital of Guangxi Zhuang Autonouous Region	South China	Guangxi Zhuang Autonomous Region
12	Yining Yang	People's Hospital of Xinjiang Uygur Autonomous Region	Northwest China	Xinjiang
13	Di Wu	Emgency General Hospital	North China	Beijing
14	Yiqiang Yuan	Henan Provincial Chest Hospital	Central China	Henan
15	Jing Wan	Zhongnan Hospital of Wuhan University	Central China	Hubei
16	Yuliang Ma	Peking university People’s hospital	North China	Beijing
17	Jun Zhang	Cangzhou Central Hospital of Tianjin Medical University	North China	Hebei
18	Zhimin Du	Dongguan Tungwah Hospital	South China	Guangdong
19	Qing Yang	Tianjin Medical University General Hospital	North China	Tianjin
20	Jinsong Cheng	Ningbo First Hospital	East China	Zhejiang
21	Chunhua Ding	Aerospace Center Hospital	North China	Beijing
22	Xiang Ma	The First Affiliated Hospital of Xinjiang Medical University	Northwest China	Xinjiang

23	Chunlin Yin	Xuanwu Hospital	North China	Beijing
24	Zeyuan Fan	Civil Aviation General Hospital	North China	Beijing
25	Qiang Tang	Peking University Shougang Hospital	North China	Beijing
26	Yue Li	the First Affiliated Hospital, Harbin Medical University	Northeast China	Heilongjiang
27	Lihua Sun	Fifth Affiliated Hospital of Xinjiang Medical University	Northwest China	Xinjiang
28	Chengzhi Lu	First Central Hospital of Tianjin	North China	Tianjin
29	Jufang Chi	Shaoxing People's Hospital	East China	Zhejiang
30	Zhuhua Yao	Tianjin Union Medicine Center	North China	Tianjin

Table S1. Baseline characteristics between the final population and population without HbA_{1c} or ABG measurements data

Characteristic	Final population *N* = 714	Population without ABG or HbA1c data	*P* value
Age, mean (SD), yrs	62.0 (10.4)	61.9 (10.8)	0.880
Male, No. (%)	535 (74.9)	321 (70.5)	0.114
SBP, mean (SD), mmHg	141.6 (24.9)	142.7 (26.2)	0.450
DBP, mean (SD), mmHg	78.3 (13.4)	80.1 (14.4)	0.026
Heart rate, mean (SD), beats/min	80.4 (15.0)	82.8 (14.9)	0.007
Medical history and risk factors, No. (%)			
Hypertension	661 (92.6)	432 (94.9)	0.139
Diabetes mellitus	450 (63.0)	175 (38.5)	<0.001
Current smoker	127 (17.8)	79 (17.4)	0.915
Atrial fibrillation	63 (8.8)	41 (9.0)	0.996
Cerebrovascular disease	139 (19.5)	88 (19.3)	1.0
Valvular disease	24 (3.4)	12 (2.6)	0.600
Peripheral arterial disease	74 (10.4)	41 (9.0)	0.511
Previous myocardial infarction	103 (14.4)	47 (10.3)	0.051
Previous intervention, No. (%)			
PCI	144 (20.2)	81 (17.8)	0.355
CABG	12 (1.7)	3 (0.7)	0.213
Dialysis modality, No. (%)			0.144
Hemodialysis	655 (91.7)	405 (89.0)	
Peritoneal dialysis	59 (8.3)	50 (11.0)	
Vintage, yrs			0.367
<1	151 (21.1)	85 (18.7)	
1-5	339 (47.5)	206 (45.3)	
5-10	183 (25.6)	131 (28.8)	
≥10	41 (5.7)	33 (7.3)	
Cause of dialysis, No. (%)			<0.001
Diabetes mellitus	267 (37.4)	96 (21.1)	
Hypertension	87 (12.2)	63 (13.8)	
Glomerulonephritis	168 (23.5)	115 (25.3)	
Other/unknown	192 (26.9)	181 (39.8)	
Index presentation, No. (%)			0.625
AMI	450 (63.0)	294 (64.6)	
Unstable angina	264 (37.0)	161 (35.4)	
Hemoglobin, g/L	105.7 (19.4)	102.5 (21.0)	0.009
Serum creatinine, mg/dl	8.9 (3.2)	8.8 (3.5)	0.554
GRACE score	157.0 [133.0, 182.0]	161.0 [136.0, 182.0]	0.375

Procedure characteristic, No. (%)			
Radial access	529 (74.1)	367 (80.7)	0.012
Extent of disease			
Any left main disease	91 (12.7)	44 (9.7)	0.131
2-vessel disease	187 (26.2)	134 (29.5)	0.250
≥3-vessel disease	426 (59.7)	245 (53.8)	0.057
Moderate or severe calcification	324 (45.4)	189 (41.5)	0.219
PCI treatment	512 (71.7)	326 (71.6)	1.000
Discharge medications, No. (%)			
Dual antiplatelet therapy	628 (88.0)	406 (89.2)	0.568
Aspirin	664 (93.0)	423 (93.0)	NA
Clopidogrel	596 (83.5)	370 (81.3)	NA
Ticagrelor	69 (9.7)	62 (13.6)	NA
ACE inhibitor or ARB	343 (48.0)	206 (45.3)	0.388
β-blocker	587 (82.2)	362 (79.6)	0.292
Calcium-channel blocker	467 (65.4)	291 (64.0)	0.657
Statin	678 (95.0)	422 (92.7)	0.151

Data are presented as mean (SD) or n (%).

ABG = admission blood glucose; ACE=angiotensin-converting enzyme; AMI = acute myocardial infarction; ARB=angiotensin receptor blocker; CABG = coronary artery bypass grafting; DBP = diastolic blood pressure; GRACE=Global Registry of Acute Coronary Events; PCI = percutaneous coronary intervention; SBP = systolic blood pressure; SHR = stress hyperglycemia ratio.

Table S2. Clinical outcomes according to tertiles of stress hyperglycemia ratio (SHR)

Characteristic	Overall *N* = 714	SHR1 (SHR ≤0.79) *N* = 238	SHR2 (0.79< SHR ≤1.10) *N* = 238	SHR3 (SHR >1.10) *N* = 238	*P* value
MACE*	345 (48.3)	100 (42.0)	98 (41.2)	147 (61.8)	<0.001
All-cause mortality	280 (39.2)	69 (29.0)	79 (33.2)	132 (55.5)	<0.001
Cardiovascular mortality	205 (28.7)	52 (21.8)	49 (20.6)	104 (43.7)	<0.001

*MACE is a composite of cardiovascular death, non-fatal myocardial infarction, and non-fatal stroke.

Table S3. Discrimination and reclassification performance of stress hyperglycemia ratio (SHR) in predicting outcomes after excluding patients with admission hemoglobin <100 g/L

	C-statistic (95% CI)	Δ C-statistic (95% CI)	*P* Value	Continuous NRI (95% CI)	*P* Value	IDI (95% CI)	*P* Value
**MACE**							
GRACE score	0.611 (0.568, 0.654)	Reference		Reference		Reference	
GRACE score + ABG	0.619 (0.576, 0.662)	0.008 (-0.004, 0.021)	0.197	0.093 (-0.089, 0.244)	0.249	0.012 (-0.001, 0.036)	0.169
GRACE score + SHR (as continuous variable)	0.646 (0.605, 0.687)	0.035 (0.009, 0.062)	0.009	0.139 (-0.035, 0.286)	0.179	0.040 (0.009, 0.077)	0.010
GRACE score + SHR (>1.08)	0.647 (0.606, 0.688)	0.036 (0.010, 0.063)	0.007	0.239 (0.106, 0.345)	0.010	0.034 (0.006, 0.074)	0.020
**All-cause mortality**							
GRACE score	0.637 (0.590, 0.684)	Reference		Reference		Reference	
GRACE score + ABG	0.659 (0.614, 0.704)	0.022 (0.001, 0.042)	0.039	0.148 (0.006, 0.328)	0.030	0.025 (0.003, 0.061)	0.020
GRACE score + SHR (as continuous variable)	0.786 (0.741, 0.831)	0.050 (0.017, 0.083)	0.003	0.170 (0.002, 0.341)	0.030	0.055 (0.015, 0.100)	<0.001
GRACE score + SHR (>1.08)	0.682 (0.637, 0.727)	0.045 (0.015, 0.075)	0.004	0.253 (0.120, 0.380)	<0.001	0.038 (0.005, 0.089)	0.020
**Cardiovascular mortality**							
GRACE score	0.638 (0.583, 0.693)	Reference		Reference		Reference	
GRACE score + ABG	0.655 (0.602, 0.708)	0.017 (-0.005, 0.039)	0.126	0.150 (-0.043, 0.347)	0.100	0.020 (-0.004, 0.056)	0.119
GRACE score + SHR (as continuous variable)	0.702 (0.649, 0.755)	0.064 (0.021, 0.106)	0.003	0.268 (0.005, 0.443)	0.030	0.071 (0.008, 0.144)	0.040
GRACE score + SHR (>1.08)	0.703 (0.654, 0.752)	0.066 (0.025, 0.106)	0.002	0.315 (0.126, 0.515)	0.020	0.056 (0, 0.138)	0.050

ABG = admission blood glucose; CI=confidence interval; GRACE=Global Registry of Acute Coronary Events; IDI = integrated discrimination improvement; NRI = net reclassification improvement; SHR = stress hyperglycemia ratio.

Supplemental Figures 1. The nonlinear association of stress hyperglycemia ratio with clinical outcomes in diabetic patients.

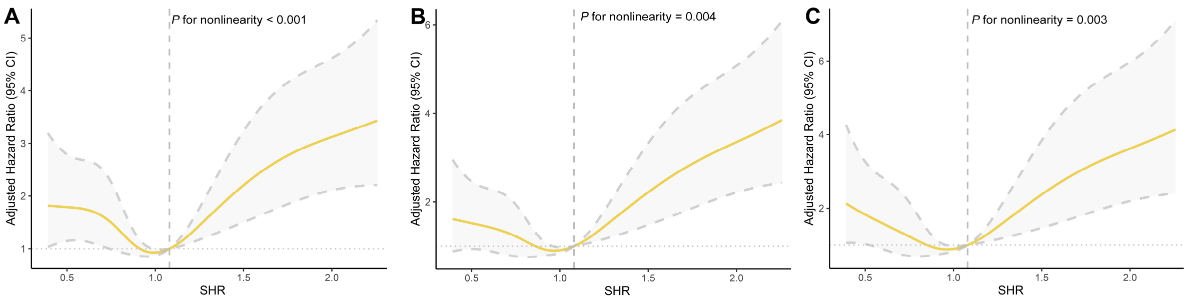

A: SHR and MACE.

B: SHR and all-cause mortality.

C: SHR and cardiovascular mortality.

Hazard ratios were indicated by yellow solid lines and 95% CIs by grey dotted line.

SHR = stress hyperglycemia ratio; CI = confidence interval.

Supplemental Figures 2. The nonlinear association of stress hyperglycemia ratio with clinical outcomes in nondiabetic patients.

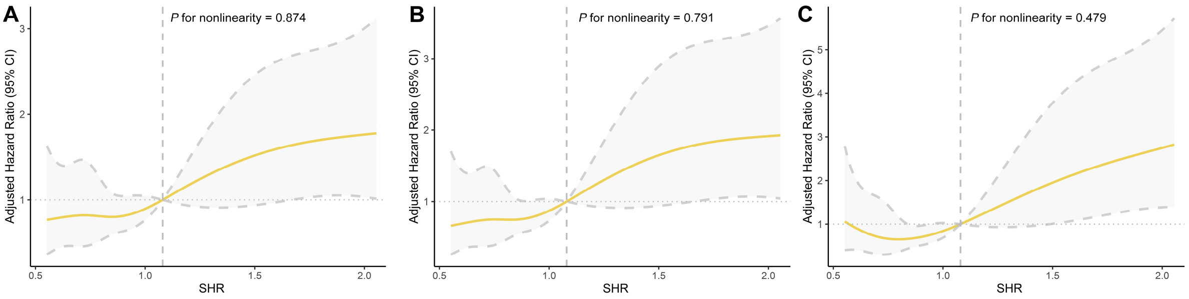

A: SHR and MACE.

B: SHR and all-cause mortality.

C: SHR and cardiovascular mortality.

Hazard ratios were indicated by yellow solid lines and 95% CIs by grey dotted line.

SHR = stress hyperglycemia ratio; CI = confidence interval.

Supplemental Figures 3. The adjusted hazards ratio of stress hyperglycemia ratio for clinical outcomes after excluding patients with admission hemoglobin <100 g/L

Outcome	HR (95% CI)	*P* value
**MACE**		
SHR	1.80 (1.38−2.35)	<0.001
First tertile of SHR	1.21 (0.84−1.74)	0.313
Second tertile of SHR	Reference	
Third tertile of SHR	2.00 (1.42−2.82)	<0.001
**All−cause Mortality**		
SHR	2.49 (1.85−3.35)	<0.001
First tertile of SHR	1.15 (0.75−1.79)	0.519
Second tertile of SHR	Reference	
Third tertile of SHR	2.73 (1.84−4.05)	<0.001
**Cardiovascular Mortality**		
SHR	2.95 (2.11−4.13)	<0.001
First tertile of SHR	1.48 (0.86−2.55)	0.156
Second tertile of SHR	Reference	
Third tertile of SHR	4.02 (2.45−6.62)	<0.001

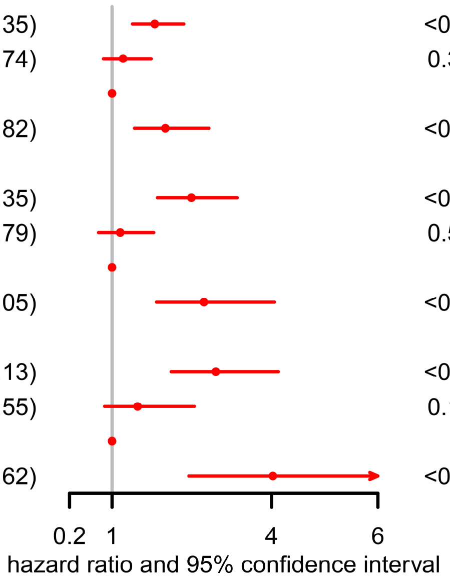

HR = hazard ratio; CI = confidence interval; SHR = stress hyperglycemia ratio.

Supplemental Figures 4. The nonlinear association of stress hyperglycemia ratio with clinical outcomes after excluding patients with admission hemoglobin <100 g/L.

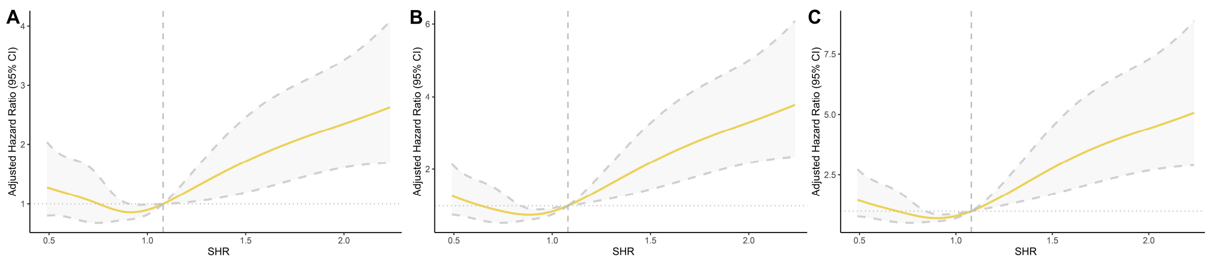

A: SHR and MACE.

B: SHR and all-cause mortality.

C: SHR and cardiovascular mortality.

Hazard ratios were indicated by yellow solid lines and 95% CIs by grey dotted line.

SHR = stress hyperglycemia ratio; CI = confidence interval.
